# Impact of *BDNF* Val66Met Polymorphism on Myocardial Infarction: Exploring the Macrophage Phenotype

**DOI:** 10.3390/cells9051084

**Published:** 2020-04-27

**Authors:** Leonardo Sandrini, Laura Castiglioni, Patrizia Amadio, José Pablo Werba, Sonia Eligini, Susanna Fiorelli, Marta Zarà, Silvia Castiglioni, Stefano Bellosta, Francis S. Lee, Luigi Sironi, Elena Tremoli, Silvia Stella Barbieri

**Affiliations:** 1 Centro Cardiologico Monzino IRCCS, 20138 Milano, Italy; leonardo.sandrini@ccfm.it (L.S.); patrizia.amadio@ccfm.it (P.A.); pablo.werba@ccfm.it (J.P.W.); sonia.eligini@ccfm.it (S.E.); susanna.fiorelli@ccfm.it (S.F.); marta.zara@ccfm.it (M.Z.); luigi.sironi@unimi.it (L.S.); elena.tremoli@ccfm.it (E.T.); 2 Dipartimento di Scienze Farmaceutiche, Università degli Studi di Milano, 20133 Milano, Italy; laura.castiglioni@unimi.it; 3 Dipartimento di Scienze Farmacologiche e Biomolecolari, Università degli Studi di Milano, 20133 Milano, Italy; silvia.castiglioni@unimi.it (S.C.); stefano.bellosta@unimi.it (S.B.); 4 Department of Psychiatry, Weill Cornell Medical College, New York, NY 10065, USA; fslee@med.cornell.edu

**Keywords:** BDNF Val66Met polymorphism, myocardial infarction, macrophage phenotype, cardiovascular disease

## Abstract

Brain-derived neurotrophic factor (BDNF) is a member of the neurotrophin growth factor family, well known for its role in the homeostasis of the cardiovascular system. Recently, the human BDNF Val66Met single nucleotide polymorphism has been associated with the increased propensity for arterial thrombosis related to acute myocardial infarction (AMI). Using cardiac magnetic resonance imaging and immunohistochemistry analyses, we showed that homozygous mice carrying the human BDNF Val66Met polymorphism (BDNF^Met/Met^) undergoing left anterior descending (LAD) coronary artery ligation display an adverse cardiac remodeling compared to wild-type (BDNF^Val/Val^). Interestingly, we observed a persistent presence of pro-inflammatory M1-like macrophages and a reduced accumulation of reparative-like phenotype macrophages (M2-like) in the infarcted heart of mutant mice. Further qPCR analyses showed that BDNF^Met/Met^ peritoneal macrophages are more pro-inflammatory and have a higher migratory ability compared to BDNF^Val/Val^ ones. Finally, macrophages differentiated from circulating monocytes isolated from BDNF^Met/Met^ patients with coronary heart disease displayed the same pro-inflammatory characteristics of the murine ones. In conclusion, the BDNF Val66Met polymorphism predisposes to adverse cardiac remodeling after myocardial infarction in a mouse model and affects macrophage phenotype in both humans and mice. These results provide a new cellular mechanism by which this human BDNF genetic variant could influence cardiovascular disease.

## 1. Introduction

Neurotrophins are a family of proteins well known for their critical role in the development, maintenance, and plasticity of the neurons [1] as well as of the heart and vasculature tissue development [2,3,4]. Among the members of this family of proteins, particular attention has been paid to the brain-derived neurotrophic factor (BDNF), a key regulator of homeostasis and pathogenesis of the cardiovascular system [5,6]. BDNF triggers the activation of endothelial cells, vascular smooth muscle cells, and monocyte/macrophages [7,8,9,10], and promotes cardiac microvasculature development and dynamics [4,11,12], suggesting its important role in orchestrating the mechanisms of repair after myocardial infarction (MI) [10].

Low circulating BDNF levels were associated with future coronary events and mortality in angina pectoris patients [13] and with heart failure biomarkers and adverse left ventricular (LV) cardiac remodeling [14]. Moreover, in different mouse models, low BDNF levels affect cardiac remodeling [15] and are associated with reduced ejection fraction and vascularization of the border zone of the infarcted region [16]. Okada et al. [16] provided evidence that these effects are independent of the cardiomyocyte-specific ablation of *BDNF* but mainly related to circulating BDNF and its cerebral output. BDNF, in addition to its ability to favor angiogenesis, promotes the shift of macrophage phenotype from M1 to M2 with consequent modification of inflammatory microenvironment [9] and, potentially, with an improvement of cardiac function after MI.

In humans, the presence of a single nucleotide polymorphism (SNP) in the *BDNF* gene, leading to valine to methionine substitution at position 66 in the prodomain region of the BDNF protein (BDNF Val66Met) determines the reduction of cerebral BDNF levels [17], and it is related to mood disorders and neurodegenerative diseases [18]. Several studies investigated the potential implications of this mutation in the context of cardiovascular diseases, but their results were controversial [19,20,21]. We showed that Met homozygosity is associated with arterial thrombosis in a knock-in mouse model and with increased risk of acute myocardial infarction (AMI) in humans [21]. Therefore, the objective of this study was to determine, in a mouse model carrying the BDNF Val66Met polymorphism, the impact of the mutation on cardiac remodeling after MI focusing on the characterization of macrophage phenotype. Besides, to support the potential relevance of the data obtained in the animal model we analyzed the macrophages spontaneously differentiated from monocytes isolated from homozygous Val or Met human carriers.

## 2. Materials and Methods

### 2.1. Animal Studies

BDNF Val66Met mice [22] were generated by heterozygous BDNF Val/Met interbreeding and offspring were genotyped by PCR analysis of tail tip-derived genomic DNA. Animal studies were in conformity with the European ethics legislation and approved by the Italian National Ministry of Health (375-2017PR and 270-2019PR). All procedures were performed in 10–12-week-old wild-type control (BDNF^Val/Val^) or BDNF^Met/Met^ mice. Surgical procedures were performed in mice anesthetized with ketamine chlorhydrate (75 mg/kg; Intervet, Segrate, Milan, Italy) and medetomidine hydrochloride (1 mg/kg; Virbac, Milan, Italy).

### 2.2. Left Anterior Descending (LAD) Coronary Artery Ligation Model

As previously described [23], MI was induced in anesthetized mice by ligation of the left anterior descending (LAD) coronary artery using a 7-0 silk suture through left thoracotomy under anesthetized mice and mechanical ventilation. Successful ligation was verified by visual inspection of the left ventricular (LV) apex for myocardial blanching, indicating interruption of the coronary blood flow. Atipamezole (0.5 mg/kg, Virbac, Milan, Italy) was administered to encourage animal awakening, and then the animals were extubated and monitored.

Based on our previous data [24], infarcted mice with a left ventricular ejection fraction (LV EF), within the range 35–45%, obtained by cardiac magnetic resonance imaging (cMRI) at 24 h, were included into the study and randomized into two different experimental protocols as follows.

Protocol 1. The impact of BDNFVal66Met polymorphism on cardiac remodeling following MI was evaluated on BDNF^Val/Val^ and BDNF^Met/Met^ mice. Mice were longitudinally examined before LAD ligation (baseline) and 24 h, 1, 4, and 8 weeks after surgery by cMRI in order to evaluate LV parameters. After the last cMRI visualization, mice were euthanized and cardiac tissue collected for histological analyses.

Protocol 2. The implication of macrophage polarization in cardiac remodeling following MI was evaluated on BDNF^Val/Val^ and BDNF^Met/Met^ sacrificed at 3, 5, and 7 days after MI.

### 2.3. Cardiac Magnetic Resonance Imaging

Mice were anesthetized with inhaled 1% isoflurane (Merial, Toulouse, France) in 100% oxygen, fixed on a holder and placed into the 3.8-cm coil. The temperature was monitored rectally. The images were acquired using a 4.7T vertical-bore MR magnet (Bruker, Germany) and a retrospective gated cine gradient echo sequence with the following parameters: Echo time (TE) 1.9 ms, repetition time (TR) 10 ms, field of view 4 × 4 cm^2^, acquisition matrix 128 × 128 pixels, slice thickness 1.3 mm, 6–8 axial slices spaced 1 mm to fully cover the LV.

The magnetic resonance images were analyzed using custom software implemented in Python environment along the lines [24] in order to obtain end-diastolic (LV EDV), end-systolic (LV ESV), and stroke volumes (LV SV); ejection fraction (LV EF); and ventricular mass (LV mass).

### 2.4. Tissue Collection and Section Preparation

In order to prepare specimens for histological analysis, the abdominal aorta was cannulated and the heart was arrested in diastole, with 2 mL of a solution of 0.1 M CdCl_2_ and 1 M KCl, and retrogradely perfused with 0.01 M phosphate saline buffer (PBS) and then with 4% (*v/v*) phosphate-buffered formalin for 10 min each. Hearts were collected and tissues post-fixed in 4% phosphate-buffered formalin for 24 h and embedded in paraffin. Consecutive 8-µm heart axial (along the apex-basis axis) sections were prepared.

### 2.5. Determination of Infarct Size and Cardiomyocytes’ Cross-Sectional Area

MI size was determined on LV axial section (one for each millimeter of LV). To define the infarct lengths, endocardial infarct length was taken as the length of endocardial infarct scar surface that included greater than 50% of the whole thickness of myocardium, and epicardial infarct length as the length of the transmural infarct region. Epicardial infarct ratio was obtained by dividing the sum of epicardial infarct lengths from all sections by the sum of epicardial circumferences from all sections. Endocardial infarct ratio was calculated similarly. Infarct size derived from this approach was calculated as ((epicardial infarct ratio + endocardial infarct ratio)/2) × 100 [25].

For collagen staining, heart sections were incubated in 0.1% Sirius red solution (Direct Red 80, Sigma-Aldrich, Saint Louis, MO, USA), mounted with DPX (Distyrene and xylene) mountant for microscopy, and images acquired with a high-resolution digital camera using 1:1 macro-lens. Myocytes’ cross-sectional area (MCSA) was measured on 3 tissue sections from each heart stained with germ agglutinin–Alexa Fluor 488, incubated with Hoechst 33258 nuclear stain (Invitrogen, Waltham, MA, USA), and then mounted using fluorescence mounting medium (Dako, Milan, Italy). Images from the non-infarcted region (222 μm x 166 μm) were acquired using a fluorescence microscope (Axiovert 200, Zeiss, Jena, Germany). Cross-sectional area of 100–150 cardiomyocytes from each mouse was measured on transversally sectioned cells with circularity greater than 0.6 and round nuclei. All quantitative analyses were performed in blind using Photoshop CS6 (Adobe System) or Image J (U.S. National Institutes of Health, Bethesda, MD, USA).

### 2.6. Cardiac Macrophage Extraction and Flow Cytometer Analysis

Hearts were collected from mice 3, 5, or 7 days after LAD ligation. Macrophages were isolated from the heart following a previously published protocol [26]. Briefly, tissue was minced in small pieces (2–3 mm length) and digested with 600 U/mL collagenase II and 10 U/mL DNase I (Sigma-Aldrich, Saint Louis, MO, USA) in Hanks buffered saline solution (Sigma-Aldrich, Saint Louis, MO, USA) and filtered through a 30-µm separation filter to generate a single-cell suspension. Macrophages were isolated after gradient centrifugation at the interphase between 35% and 70% Percoll solutions (Sigma-Aldrich, Saint Louis, MO, USA), then washed with PBS, permeabilized, and stained with the following fluorophore-conjugated antibodies: CD206-APC (eBioscience-Thermo Fisher, Paisley, Scotland, UK), CD68-PE (eBioscience-Thermo Fisher, Paisley, Scotland, UK), and CD11c-FITC (eBioscience-Thermo Fisher, Paisley, Scotland, UK). Nonspecific staining was eliminated, incubating the same number of cells as in the surface markers’ antibody tube with isotype control antibody, matched to the surface marker antibody’s host species and class. Samples were analyzed by NovoCyte flow cytometer (ACEA Biosciences, San Diego, CA, USA) and the percentage of positive cells was compared with fluorescence-labeled isotype controls. A minimum of 10,000 events was collected in the CD68^+^ gate by flow cytometry.

### 2.7. Mouse Peritoneal Macrophage Isolation

Seventy-two hours after thioglycollate broth injection, macrophages were harvested from the peritoneum with ice-cold PBS, centrifuged, and suspended in DMEM (Dulbecco’s Modified Eagle Medium) medium (Lonza, Basel, Switzerland) supplemented with penicillin and streptomycin (100 U/mL each) (Gibco, Rodano, Milan, Italy) and plated as previously described [27]. After one hour, plates were washed and adherent cells were left for four hours in DMEM medium after which they were lysed TRIzol Reagent (Sigma-Aldrich, Saint Louis, MO, USA) or within RIPA (Radioimmunoprecipitation assay) buffer (1% Triton X-100 in 50 mM Tris–HCl (pH 7.5) plus 1 mM phenylmethylsulfonyl fluoride, 1 mM N-ethylmaleimide, 2 μg/mL leupeptin, 1 mM NaF, 1 mM benzamidine, and 1 mM sodium vanadate, all from Sigma-Aldrich, Saint Louis, MO, USA. After incubation on ice for 1 h, the lysates were centrifuged at 12,000 × *g* for 30 min for BDNF detection and frozen for further analyses.

### 2.8. BDNF Analysis

BDNF was measured by commercial Emax Immunoassay system kit (Promega, Madison, WI, USA) in whole macrophages’ extract lysed in RIPA buffer.

### 2.9. Migration Assay

Boyden chamber assay. Cell migration under unstimulated conditions and in response to a migratory factor was assessed by using a 48-well microchemotaxis Boyden chamber [28]. Briefly, triplicate wells of the lower part of the microchemotaxis chamber were filled with DMEM (Lonza, Basel, Switzerland) with 0.2% BSA (Bovine Serum Albumin) ± 20 ng/mL MCP-1 (Monocyte Chemoattractant Protein-1) (Thermo Fisher, Paisley, Scotland, UK). A 5-µm pore diameter polycarbonate membrane coated with collagen was placed on the top and the chamber was tightened. Peritoneal macrophages (1 × 10^5^ cells) were added to the upper wells for 3 h at 37 °C. The membrane was then removed, adherent cells on the top were eliminated, and the membrane was stained with Diff-Quik reagents. Each test group was assayed in at least 3 replicates and pictures of typical fields were taken under high-power light microscopy. Migrated cells were evaluated by blind counting cells in 12 random fields for each experimental group with the Image J software.

The agarose spot migration assay was adapted from Ahmed et al. [29]. Ten µL of low-gelling temperature agarose (Sigma-Aldrich, Saint Louis, MO, USA) containing vehicle, LPS (Lipopolysaccharide) (10 ng/mL), or MCP-1 (20 ng/mL) (Thermo Fisher, Paisley, Scotland, UK) were put on the surface of a microscope glass slide coated with gelatin. Then, 5 × 10^5^ macrophages were seeded on every microscope glass slide with DMEM medium (Lonza, Basel, Switzerland) supplemented with penicillin and streptomycin (100 U/mL each), non-adherent cells were washed with PBS after 1 h, and macrophages were allowed to migrate for 24 h in DMEM medium (Lonza, Basel, Switzerland) supplemented with penicillin and streptomycin (100 U/mL each) (Gibco, Rodano, Milan, Italy) and 10% of serum (Euroclone, Pero, Milan, Italy). Photos were taken using a 5 × magnification with a Zeiss Axioskop (Zeiss, Jena, Germany) equipped with an intensified charge-coupled device (CCD) camera system (Photometrics, Tucson, AZ, USA). The number of migrated macrophages was determined by blind counting cells in 8 random fields for each experimental group.

### 2.10. Human Studies

Homozygous BDNF^Val/Val^ and BDNF^Met/Met^ carriers (age 50–80 y) were identified within a large set of male patients with a clinical history of stable angina or acute coronary syndrome followed by coronary artery bypass graft surgery, previously genotyped by our group for the BDNF Val66Met polymorphism [21]. Patients were matched for genotype, age clinical presentation of coronary heart disease as stable and unstable angina or acute myocardial infarction, and pharmacological treatments. All the patients were under aspirin and statins, one patient per group was under anticoagulant, and 92% of BDNF^Val/Val^ patients and 71% of BDNF^Met/Met^ patients were under beta-blockers. After selected candidates provided written informed consent, a detailed anamnesis and physical examination were carried out and venous blood was obtained at fasting from an antecubital vein. The study complied with the declaration of Helsinki and was approved by the Institutional Review Board and Ethical Committee of Centro Cardiologico Monzino IRCCS (Istituto di ricovero e cura a carattere scientifico) (R455/16-CCM 471). Human mononuclear cells were separated from venous blood by centrifugation on Ficoll-Paque Plus (Sigma-Aldrich, Saint Louis, MO, USA) density gradient, and monocytes were isolated by selective adhesion [30]. Monocyte to macrophage differentiation was obtained by cultivating monocytes in Medium 199 (Lonza, Basel, Switzerland) supplemented with l-glutamine, penicillin, and streptomycin (all from Gibco, Rodano, Milan, Italy), and 10% of autologous serum at 37 °C and 5% CO_2_ for seven days, as previously described [31].

### 2.11. Quantitative Real-Time PCR (qPCR)

Total cellular RNA was isolated from human and murine macrophages using TRIzol Reagent (Sigma-Aldrich, Saint Louis, MO, USA) and a Direct-zol RNA extraction kit (Zymo Research, Irvine, CA, USA) according to the manufacturer’s instructions. One μg of RNA was reverse transcribed using the iScript Advanced cDNA Synthesis Kit (Bio-Rad Laboratories, Segrate, Milan, Italy).

Samples of cDNA were incubated in 15 µL Luna® Universal qPCR mix containing the specific primers and fluorescent dye SYBR Green (New England Biolabs, Pero, Milan, Italy). RT-qPCR was carried out in triplicate for each sample on the CFX Connect real-time System (Bio-Rad Laboratories, Segrate, Milan, Italy), as previously described [32]. Gene expression was analyzed using parameters available in CFX Manager Software 3.1 (Bio-Rad Laboratories, Segrate, Milan, Italy). Then, qPCR was carried out using the primer sequences shown in Table S1.

### 2.12. Statistical Analyses

Statistical analyses were performed using GraphPad Prism 7 software (San Diego, CA, USA). Data were analyzed by Student’s t-test or by 2-way-ANOVA (with repeated measures when necessary) for main effects of treatment, time, and genotype, followed by a Bonferroni post hoc analysis. *p* values of less than 0.05 were considered statistically significant. Data represent mean ± SEM. All human performed analyses were adjusted for age and drug treatments.

## 3. Results

### 3.1. BDNF Val66Met Polymorphism Affects Left Ventricle Remodelling After LAD Ligation

To establish the effect of BDNF Val66Met polymorphism in heart remodeling after MI, the BDNF^Val/Val^ and homozygous BDNF^Met/Met^ mice underwent left anterior descending (LAD) coronary artery ligation, and cardiac morphology and function were assessed by cMRI at 24 h and 1, 4, and 8 weeks after surgery. At baseline, BDNF^Val/Val^ and BDNF^Met/Met^ mice displayed normal and comparable heart parameters (Table 1).

**Figure 1:**
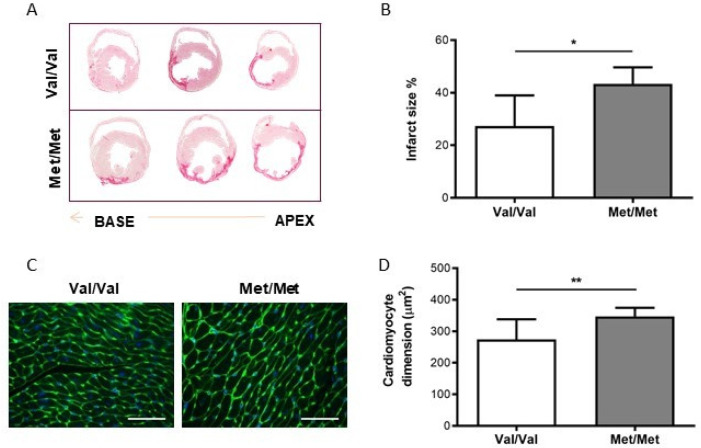
Characterization of the infarcted hearts isolated from BDNF^Val/Val^ (Val/Val) and BDNF^Met/Met^ (Met/Met) mice. (**A**) Representative photomicrographs of infarct size evaluated from the base (left) to the apex (right) by Sirius red staining measured at 8 weeks after surgery. (**B**) Infarct size expressed as percentage of the length of the infarct scar on the LV total circumferential length. (**C**) Representative immunofluorescence (40×, scale bar: 50 µm) and (**D**) measurement of myocyte cross-sectional area (MCSA) in the remote left ventricle (LV). *N* = 6/group, * *p* < 0.05, ** *p* < 0.01.

As a consequence of MI, 24 h after LAD ligation an important reduction of left ventricular ejection fraction (LV EF%) was observed in both experimental groups (BDNF^Val/Val^: −45 ± 2%, BDNF^Met/Met^: −47 ± 2%). Although BDNF^Met/Met^ mice showed an increase of left ventricle end-diastolic and systolic volumes compared to BDNF^Val/Val^ group (LV EDV: BDNFV^al/Val^: +27 ± 5%; BDNF^Met/Met^: +70 ± 7% and LV ESV: BDNF^Val/Val^: +149 ± 17%; BDNF^Met/Met^: +294 ± 48%), no significant difference in the cMRI parameters was detected either at 24 h or 1 week after LAD ligation. Four weeks after surgery, BDNF^Met/Met^ mice displayed a significant increase in LV EDV and LV ESV compared to BDNF^Val/Val^ (*p* < 0.05 for LV EDV and *p* < 0.01 for LV ESV) with a concurrent LV EF% reduction (*p* < 0.05). The LV EDV and the LV ESV differences were maintained until 8 weeks after LAD ligation, when also a significant increase of LV mass was observed in BDNF^Met/Met^ (*p* < 0.05) (Table 1). At the same time point, histological examination showed that BDNF^Met/Met^ mice have a larger infarct size % (*p* < 0.05) (Figure 1A,B) and a greater myocyte cross-sectional area (MCSA) in non-infarcted left ventricular regions (*p* < 0.05) than BDNF^Val/Val^ mice (Figure 1C,D).

### 3.2. BDNF Val66Met Polymorphism Affects the Physiological Accumulation of M1- and M2-Like Macrophages after LAD Ligation

Ischemia triggers the accumulation of macrophages that plays a fundamental role in post-MI damage and in the subsequent cardiac remodeling [33]. Indeed, it is well known that the macrophage phenotype regulates the inflammatory phase and influences the reparative phase favoring collagen deposition and scar formation. Then, we profiled macrophages’ accumulation in the myocardium and we investigated the cardiac macrophage subsets at 3, 5, and 7 days after MI.

**Figure 2:**
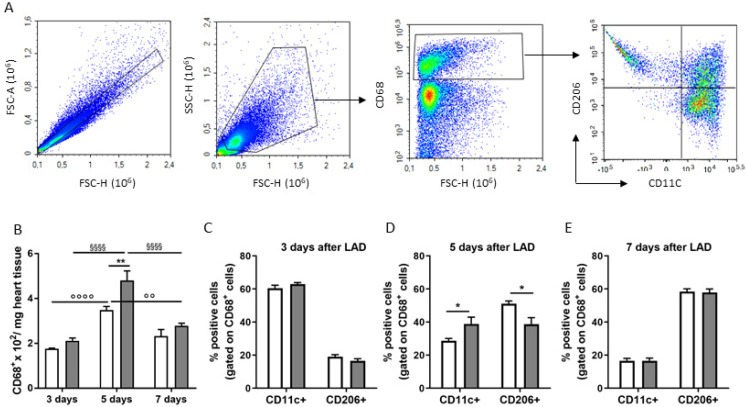
Flow cytometry analyses (FACS) of macrophages isolated at a different time point (3, 5, and 7 days) after the induction of myocardial infarction in BDNF^Val/Val^ (white bar graph) and BDNF^Met/Met^ (grey bar graph) mice. (**A**) Representative FACS plots showing the gating strategy for CD11c^+^ (CD11c^+^/CD68^+^) and CD206^+^ (CD206^+^/CD68^+^). Quantification of macrophage subsets according to the expression of (**B**) CD68 positive cells, CD206, and CD11c positive cells at (**C**) 3, (**D**) 5, (**E**) 7 days after LAD. *N* = 6/group/time; * *p* < 0.05 and ** *p* < 0.01 BDNF^Val/Val^ vs. BDNF^Met/Met^; °° *p* < 0.01 and °°°° *p* < 0.001 BDNF^Val/Val^ comparison at different time points; ^§§§§^ *p* < 0.001 BDNF^Met/Met^ comparison at different time points.

The infarct induced, in both BDNF^Val/Val^ and BDNF^Met/Met^ mice, the accumulation of macrophages (CD68^+^ cells), which peaked on day 5. Of note, at day 5, infarcted BDNF^Met/Met^ heart accumulated a greater number of macrophages compared to BDNF^Val/Val^ (Figure 2A,B), suggesting a prolonged recruitment of macrophages in the heart of mutant mice.

According to the literature, we found a progressive reduction in classically activated macrophages (CD11c^+^/CD68^+^, pro-inflammatory M1-like) associated with a concomitant increase in alternatively activated macrophage markers (CD206^+^/CD68^+^, M2-like) from day 3 to day 7 after LAD ligation (Figure 2C–E) in BDNF^Val/Val^ mice.

Remarkably, on day 5, BDNF^Met/Met^ mice still showed a persistent presence of CD11c^+^/CD68^+^ macrophages and, of consequence, a reduced accumulation of CD206^+^/CD68^+^ macrophages, compared to BDNF^Val/Val^ (Figure 2C–E).

### 3.3. BDNF Mutation Influences Murine Macrophage Polarization

To assess whether the BDNF Val66Met affects macrophage polarization, in vitro studies were performed in resident and thioglycollate-elicited peritoneal macrophages isolated from BDNF^Val/Val^ and BDNF^Met/Met^ mice.

**Figure 3:**
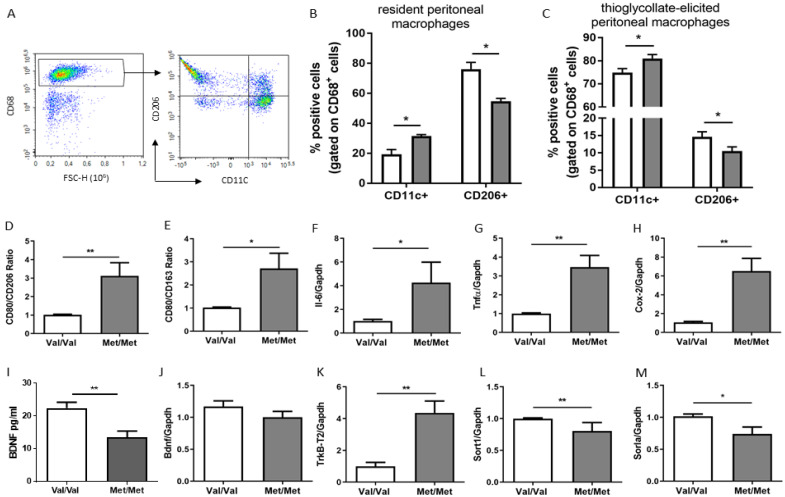
Characterization of peritoneal macrophages isolated from BDNF^Val/Val^ (Val/Val, white bar graph) and BDNF^Met/Met^ (Met/Met, grey bar graph) mice. (**A**) Representative FACS plots showing the gating strategy for CD11c^+^ (CD11c^+^/CD68^+^) and CD206^+^ (CD206^+^/CD68^+^). Quantification of (**B**) resident and (**C**) thioglycolate-elicited peritoneal macrophage according to the expression of CD206 and CD11c positive cells. The mRNA expression levels of genes related to (**D**,**E**) classically/alternatively activated macrophage marker ratio, (**F**,**G**,**H**) inflammatory genes (*Il-6, Tnf-α,* and *Cox-2*), (**J**) *Bdnf*, and (**K**) *TrkB-T2*, and (**L**,**M**) genes involved in its sorting (*Sort1* and *Sorla*). (**I**) BDNF levels detected by ELISA assay. *N* = 6 mice/group, * *p* < 0.05, and ** *p* < 0.01.

Flow cytometry analysis revealed a higher number of CD11c^+^ and lower abundance of CD206^+^ in cells isolated from BDNF^Met/Met^ when compared to BDNF^Val/Val^ in both resident and thioglycolate-elicited macrophages (Figure 3A–C). In addition, BDNF^Met/Met^ thioglycolate-elicited peritoneal macrophages expressed significantly higher levels of marker CD80 (BDNF^Val/Val^: 1.03 ± 0.02 vs. BDNF^Met/Met^: 1.35 ± 0.16, *p* < 0.05) and lower levels of alternatively activated macrophage markers CD206 (BDNF^Val/Val^: 1.02 ± 0.01 vs. BDNF^Met/Met^: 0.63 ± 0.16, *p* < 0.05) and CD163 (BDNF^Val/Val^: 1.00 ± 0.001 vs. BDNF^Met/Met^: 0.58 ± 0.08, *p* < 0.001), with an increase in the CD80/CD206 and CD80/CD163 ratio (Figure 3A,B). The abnormal ratio in classically/alternatively activated macrophage markers detected in mutant macrophages was associated with a greater expression of inflammatory cytokines (Figure 3F–H) that well reflected their predominant pro-inflammatory (M1-like) phenotype.

Interestingly, BDNF Val66Met polymorphism reduced the levels of BDNF in macrophages (Figure 3I) without affecting its mRNA levels (Figure 3J), and increased the expression of its specific receptor *TrkB-T2* (Figure 3K). In addition, a significant reduction of both *Sort1* and *Sorl1*, known to mediate the trafficking and sorting of TrkB receptors of BDNF, were found in mutant macrophages (Figure 3L,M).

### 3.4. BDNFVal66Met Polymorphism Affects Murine Macrophages’ Shape and Migratory Ability

**Figure 4:**
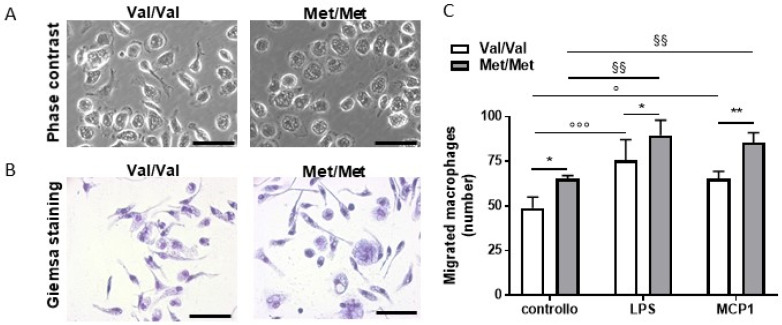
Morphology and functionality of BDNF^Val/Val^ (Val/Val) and BDNF^Met/Met^ (Met/Met) peritoneal macrophages. (**A**) Representative images of phase-contrast microscopy and (**B**) Giemsa staining, 40×, scale bar: 50 µm. (**C**) Migratory ability analyzed by agarose spot migration assay. *N* = 4 independent experiments; * *p* < 0.05, and ** *p* < 0.01 Val/Val vs. Met/Met; ° *p* < 0.05 and °°° *p* < 0.005 vs. BDNF^Val/Val^ control macrophages; ^§§^ *p* < 0.001 vs. BDNF^Met/Met^ control macrophages.

As previously described in several studies [34], here we observed that the pro-inflammatory macrophage phenotype was associated with morphological and functional changes. In particular, the microscopic examination of macrophages showed that cells isolated from BDNF^Met/Met^ mice exhibited mainly a round morphotype compared to BDNF^Val/Val^ that, in turn, were prevalently spindle/elongated (Figure 4A,B).

In addition, in vitro cell migration studies showed that elicited BDNF^Met/Met^ macrophages displayed a higher migratory ability compared to BDNF^Val/Val^ (Figure 4C and Figure S1). This increase still remained significantly different both in response to MCP-1 and after LPS stimulation, providing evidence of a broad migratory alteration induced by the presence of this mutation (Figure 4C and Figure S1).

### 3.5. Effect of BDNFVal66Met Polymorphism on Human Macrophages Phenotype

Finally, we compared the phenotype of monocyte-derived macrophages isolated from BDNF^Val/Val^ and BDNF^Met/Met^ coronary artery disease (CAD) patients representing the subset of the same cohort of CAD patients previously analyzed [21].

**Figure 5:**
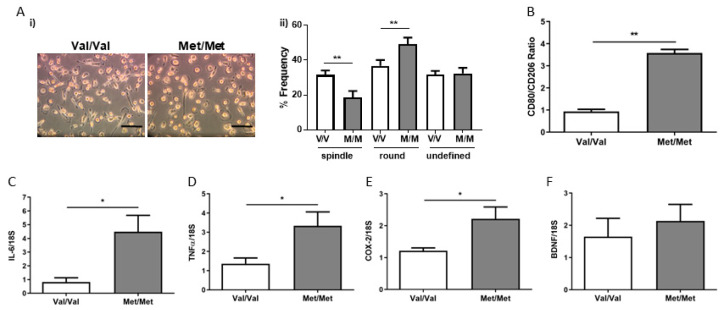
Characterization of BDNF^Val/Val^ (Val/Val, white bar graph) and BDNF^Met/Met^ (Met/Met, grey bar graph) human macrophages. (**A**) (i) Bright field representative images (20×, scale bar: 50 µm) and (ii) frequency in round, spindle, and undefined human macrophages. (**B**) mRNA levels of genes related to M1/M2 markers ratio, (**C**–**E**) inflammatory genes (*IL-6, TNF-α,* and *COX-2*) and (**F**) *BDNF*. *N* = 13/14 patient genotype/group, * *p* < 0.05, and ** *p* < 0.01.

As observed in mouse peritoneal macrophages, the macrophage obtained from BDNF^Met/Met^ subjects showed a higher percentage of round morphotype, while BDNF^Val/Val^ macrophages had a prevalently elongated/spindle shape (Figure 5A).

In addition, in the presence of the mutation, we observed an increase in the transcript level of *CD80* (BDNF^Val/Val^: 1.00 ± 0.26; BDNF^Met/Met^: 3.52 ± 0.87, *p* < 0.05) and lower levels of *CD206* (BDNF^Val/Val^: 1.10 ± 0.20; BDNF^Met/Met^: 0.28 ± 0.08, *p* < 0.01) with an increase in the *CD80/CD206* ratio (Figure 5B). Finally, BDNF^Met/Met^ human macrophages expressed a significantly higher level of inflammatory genes (Figure 5C–E). As showed above in mouse macrophages, the *BDNF* mRNA levels were similar between Met and Val carrier patients (Figure 5F).

## 4. Discussion

Despite the advances in prevention and treatment, cardiovascular diseases remain the leading cause of morbidity and mortality in the world, with an estimated 23.3 million deaths by 2030 [35]. Among the pathologies, myocardial infarction represents a major burden since it might result in heart failure if it does not prove fatal immediately.

In the present study, using the humanized BDNF Val66Met homozygous knock-in mice, we found that the BDNF Val66Met polymorphism predisposes to adverse cardiac remodeling with a persistent presence of pro-inflammatory macrophages and a reduced accumulation of reparative macrophage phenotype in the infarcted heart.

The macrophage population in mouse ischemic heart was heterogeneous and changes during the time after the event. In particular, pro-inflammatory (M1) macrophages were dominant at day 1–3 post-MI, whereas reparative (M2) macrophages represented the majority between days 5–7 [36]. Indeed, the pro-inflammatory M1 macrophages played a key role in the first step after damage, releasing several inflammatory factors, including cytokines, chemokines, growth factors, and Matrix metallopeptidases (MMPs), in order to clear the damaged area from cell debris and degrade the extracellular matrix [37]. The anti-inflammatory M2 macrophages were involved in the reparative processes, they produced anti-inflammatory, pro-angiogenic, and pro-reparative factors in order to facilitate neo-angiogenesis and scar repair [38].

The correct temporal window during which pro-inflammatory or reparative macrophages are present in the infarcted area is fundamental for the correct process of cardiac remodeling, improving myocardial repair, and function post-MI [37]. The persistence of pro-inflammatory macrophages for a prolonged period can lead to the expansion of infarct size and to a delayed resolution of inflammation [39]. Thus, extensive matrix degradation, compromised ventricular wall integrity, cardiac rupture, and fibrosis are followed by impaired systolic function, chamber dilation, and ventricular hypertrophy [40]. Therefore, the adverse cardiac remodeling observed in BDNF^Met/Met^ mice could well be traced back to the persistent presence of pro-inflammatory, rather than reparative, macrophages after MI. However, we cannot exclude that other leukocyte populations might affect adverse cardiovascular remodeling in BDNF^Met/Met^ mice. Here, we showed that mutation improves M1-like polarization. However, at day 3 post-MI, no difference between the two groups was observed, suggesting that the presence of a highly inflammatory cardiac environment has the same impact on the polarization of both BDNF^Val/Val^ and BDNF^Met/Met^ macrophages. In addition, mutation prevents an appropriate M2-like polarization. Interestingly at day 5 post-MI (when M2 phenotype starts to be favored), the difference between genotypes occurs, suggesting a likely defect to induce a proper M2 polarization. Specific studies must be carried out to unravel this important issue.

Interestingly, a similar macrophage shift was recently observed also in the epidydimal adipose tissue of BDNF^Met/Met^ mutant mice, where macrophages expressed higher levels of pro-inflammatory marker CD80 compared to BDNF^Val/Val^ mice [32], suggesting that this mutation might predispose macrophages to a pro-inflammatory phenotype in different tissues and experimental conditions.

In line with the in vivo model, ex vivo studies showed that the Met mutation predisposes to M1-like phenotype. Indeed, according to the definition of M1 or M2 macrophage phenotype [41], both human and mouse BDNF^Met/Met^ macrophages displayed a higher ratio between CD80 and CD163 and/or CD206 markers, greater levels of pro-inflammatory cytokines *IL-6* and *TNF-α*, and lower levels of *TGF-β* and *IL-10*, and increased migratory ability than BDNF^Val/Val^ macrophages. In addition, the abnormal ratio between round and spindle macrophages observed in Met carriers well reflects this phenotype. Actually, round macrophages are characterized by genes encoding for the pro-inflammatory cytokines and chemokines (CCR2^+^), showing an M1-like pro-inflammatory phenotype. On the contrary, spindle-shaped macrophages overexpress IL-10 and TGFβ, typical of M2 anti-inflammatory/reparative (CCR2^−^) macrophages, and possess wound repair ability [42].

After MI, the damaged cardiac tissue was able to initiate an influx of CCR2^+^ macrophages that produce pro-inflammatory cytokines [43,44], displaying an M1-like phenotype. Interestingly, suppression of the CCR2 receptor activity by specific siRNA and inhibition of CCR2^+^ macrophage recruitment in the ischemic area significantly reduced infarct size in mouse models and concomitantly decreased *MCP-1*, *IL-1-β*, *IL-6*, and *TNF-α* expression [39,42,45]. In contrast, depletion of resident cardiac CCR2- macrophages in a murine model of myocardial infarction increased infarct area, reduced LV systolic function, and exaggerated LV remodeling [42]. Therefore, the migration of circulating monocytes and resident macrophages to the damaged area plays a key role in the subsequent cardiac remodeling. In this regard, future studies will be necessary to establish the specific contribution of circulating monocytes and resident macrophages as well as the role of the cardiac environment in this animal model.

For the first time, we provided evidence that the BDNF Val66Met polymorphism affects the macrophage phenotype in both an animal model and in humans. In particular, macrophages spontaneously differentiated from BDNF^Met/Met^ patients displayed an inflammatory M1-like phenotype, despite that the patients were under treatment with statins. Indeed, it is well known that these compounds, beyond their strong lipid-lowering effect, polarize the macrophages’ phenotype toward M2, decreasing their inflammatory profile [46,47,48]. Thus, our data suggest that statin treatment may not be sufficient to counteract the inflammatory macrophage phenotype of BDNF^Met/Met^ patients. Based on these data, we can speculate that a phenotypic alteration in BDNF^Met/Met^ macrophages might affect the onset and progression of cardiovascular disease; although, it will be important to confirm all the modifications observed in mutant mouse macrophages also in human.

## 5. Conclusions

To our knowledge, these results provide new insights into the already well-established role of BDNF in the cardiovascular system, suggesting a new cellular mechanism by which the BDNF Val66Met polymorphism could affect pathological cardiovascular conditions. Further studies are necessary to transfer this information into daily clinical practice.

## Figures and Tables

**Table 1:** Left ventricular (LV) parameters obtained by cardiac magnetic resonance imaging (cMRI) analysis.

Genotype	Time	Cardiac Magnetic Resonance Imaging (cMRI) Parameters			
		LV EF (%)	LV EDV (µL)	LV ESV (µL)	LV Mass (mg)
BDNF^Val/Val^	baseline	67.5 ± 1.7	47.2 ± 3.3	15.6 ± 1.7	82.3 ± 3.1
	24 h	37.2 ± 1.8	59.9 ± 4.7	37.4 ± 2.8	72.2 ± 3.8
	1 week	36.9 ± 1.8	71.7 ± 5.9	45.1 ± 3.9	88.0 ± 6.5
	4 weeks	36.1 ± 2.2	92.1 ± 4.5	59.1 ± 4.2	84.8 ± 3.3
	8 weeks	31.8 ± 2.9	91.0 ± 6.6	62.7 ± 6.3	89.2 ± 3.8
BDNF^Met/Met^	baseline	69.9 ± 2.4	43.7 ± 2.3	13.5 ± 1.7	80.8 ± 3.5
	24 h	36.2 ± 1.1	73.1 ± 1.8	46.7 ± 1.7	82.8 ± 3.5
	1 week	33.5 ± 3.1	97.5 ± 7.2	66.5 ± 7.1	97.6 ± 3.1
	4 weeks	26.0 ± 2.4 *	128.0 ± 12.9 *	96.9 ± 11.8 **	95.6 ± 3.8
	8 weeks	25.5 ± 2.5	140.2 ± 13.3 ***	107.1 ± 13.0 ***	104.1 ± 3.8 *

BDNF^Val/Val^: *N* = 7 mice, BDNF^Met/Met^: *N* = 10 mice; LV: left ventricular; EF: ejection fraction; EDV: end-diastolic volume; ESV: end-systolic volume. Data are expressed as mean ± SEM. Two-way ANOVA followed by Bonferroni post-hoc analysis, * *p* < 0.05, ** *p* < 0.01, and *** *p* < 0.001 vs BDNF^Val/Val^.

## Supplementary Materials

The following are available online at https://www.mdpi.com/2073-4409/9/5/1084/s1. Figure S1: Migratory ability of peritoneal macrophages in Boyden chamber assay. Table S1: Primer sequences of the analyzed genes.
