# Supplementary material for: Impact of BDNF Val66Met Polymorphism on Myocardial Infarction: Exploring the Macrophage Phenotype
Source: Cells. 2020 Apr 27;9(5):1084. doi: 10.3390/cells9051084 (PMC7290372; doi:10.3390/cells9051084)
Supplement: Supplementary file 1 [file cells-09-01084-s001.zip › Supplemental Figure S1.docx]

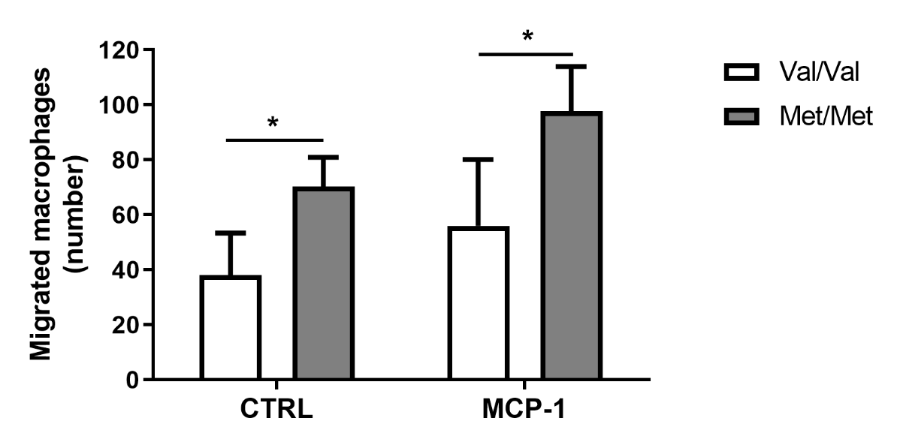


**Supplemental Fig. S1.** Migratory ability of peritoneal macrophages in Boyden chamber assay. Data are expressed as mean ± SEM. n= 4 independent experiments. Student’s t-test. * p < 0.05.
