# Supplementary material for: Impact of BDNF Val66Met Polymorphism on Myocardial Infarction: Exploring the Macrophage Phenotype
Source: Cells. 2020 Apr 27;9(5):1084. doi: 10.3390/cells9051084 (PMC7290372; doi:10.3390/cells9051084)
Supplement: Supplementary file 1 [file cells-09-01084-s001.zip › Supplemental Table S1.docx]

| MOUSE |  |  |
| --- | --- | --- |
| Gene |  |  |
| Gapdh | Fwd | CGTGCCGCCTGGAGAAACC |
|  | Rev | TGGAAGAGTGGGAGTTGCTGTTG |
| Il-6 | Fwd | CAAAGCCAGAGTCCTTCAGAG |
|  | Rev | TGGTCCTTAGCCACTCCTTC |
| TrkB-T2 | Fwd | CAGAGGACAAACATAAGCTTCACCG |
|  | Rev | TCTAACCCCCACCCTGCTC |
| Tnf-α | Fwd | TGCCTATGTCTCAGCCTCTTC |
|  | Rev | GAGGCCATTTGGGAACTTCT |
| Sorla | Fwd | GTGTGAGGACGGCGAGGCAT |
|  | Rev | GGTGGACTGCTGCCTCTGGTCA |
| Sort1 | Fwd | GCCTGTGGGTGTCCAAGAAT |
|  | Rev | GGCACCAAGATCAGCTTTGC |
| CD80 | Fwd | CTGGGAAAAACCCCCAGAAG |
|  | Rev | TGACAACGATGACGACGACTG |
| CD206 | Fwd | GTCTGAGTGTACGCAGTGGTTGG |
|  | Rev | TCTGATGATGGACTTCCTGGTAGCC |
| CD163 | Fwd | GGGTCATTCAGAGGCACACTG |
|  | Rev | CTGGCTGTCCTGTCAAGGCT |
| Cox-2 | Fwd | AGTCTCTCAATGAGTACCGCAAA |
|  | Rev | AGTTCTTCAAATGATGTGTACGG |

**Supplemental Table S1**. Primer sequences of the analyzed genes.

| HUMAN |  |  |
| --- | --- | --- |
| Gene |  |  |
| 18S | Fwd | CGGCTACCACATCCAAGGAA |
|  | Rev | CCTGTATTGTTATTTTTCGTCACTACCT |
| IL-6 | Fwd | AAATTCGGTACATCCTCGACGGCA |
|  | Rev | AGTGCCTCTTTGCTGCTTTCACAC |
| TNF-α | Fwd | CAGCCTCTTCTCCTTCCTGAT |
|  | Rev | GCCAGAGGGCTGATTAGAGA |
| CD80 | Fwd | CTCCCATCCTGGGCCATTAC |
|  | Rev | CTCTCTGCATCTTGGGGCAA |
| CD206 | Fwd | TTCGGACACCCATCGGAATTT |
|  | Rev | CACAAGCGCTGCGTGGAT |
| COX-2 | Fwd | TTAACATTGATCTGCTGACAAAACC |
|  | Rev | ACACATTTGTCTGAGGCACTGAA |
